# Supplementary material for: HIVprotI: an integrated web based platform for prediction and design of HIV proteins inhibitors
Source: J Cheminform. 2018 Mar 9;10:12. doi: 10.1186/s13321-018-0266-y (PMC5845081; doi:10.1186/s13321-018-0266-y)
Supplement: Supplementary file 2 — Additional file 2. Source code of HIVProtI web server. [file 13321_2018_266_MOESM2_ESM.zip › HIVprotI_Source-code/hivprotI/weka-3-6-6/doc/weka/filters/package-summary.html]

weka.filters


---


|  |  |  |
| --- | --- | --- |
| **Overview** \| **Package** \| Class \| **Tree** \| **Deprecated** \| **Index** \| **Help** \| **Weka's home** | |  |
| **PREV PACKAGE**   **NEXT PACKAGE** | **FRAMES**    **NO FRAMES**     **All Classes** |  |


---

## Package weka.filters

| **Interface Summary** | |
| --- | --- |
| **Sourcable** | Interface for filters that can be converted to Java source. |
| **StreamableFilter** | Interface for filters can work with a stream of instances. |
| **SupervisedFilter** | Interface for filters that make use of a class attribute. |
| **UnsupervisedFilter** | Interface for filters that do not need a class attribute. |

| **Class Summary** | |
| --- | --- |
| **AllFilter** | A simple instance filter that passes all instances directly through. |
| **CheckSource** | A simple class for checking the source generated from Filters implementing the `weka.filters.Sourcable` interface. |
| **Filter** | An abstract class for instance filters: objects that take instances as input, carry out some transformation on the instance and then output the instance. |
| **MultiFilter** | Applies several filters successively. |
| **SimpleBatchFilter** | This filter is a superclass for simple batch filters. |
| **SimpleFilter** | This filter contains common behavior of the SimpleBatchFilter and the SimpleStreamFilter. |
| **SimpleStreamFilter** | This filter is a superclass for simple stream filters. |

---


|  |  |  |
| --- | --- | --- |
| **Overview** \| **Package** \| Class \| **Tree** \| **Deprecated** \| **Index** \| **Help** \| **Weka's home** | |  |
| **PREV PACKAGE**   **NEXT PACKAGE** | **FRAMES**    **NO FRAMES**     **All Classes** |  |


---
